# Eliminating bias: a routine commitment

**DOI:** 10.1016/j.lanepe.2022.100351

**Published:** 2022-03-08

**Authors:** The Lancet Regional Health– Europe

On International Women's Day—March 8, 2022—we must reinforce that equity and career development are persistently threatened by gender bias. Worldwide, the number of women with Bachelors and Masters degrees outnumbers men, but this difference disappears at the doctoral level, whereby 57% of graduates are men and this gap widens further up the career ladder. Over the past decade, the European Union (EU) has narrowed the gender gap among doctoral graduates by only 0.6%, from 47.5% in 2010 to 48.1% in 2018. Women remain grossly underrepresented in the science, technology, engineering, and mathematics (STEM) fields. This underrepresentation of women needs to be addressed, because the presence of gender parity at the doctoral graduate level and higher is fundamental to subsequently achieving a gender-balanced research environment.

In 2019, men accounted for approximately 70% of researchers in western Europe, in contrast to 57% of researchers in countries in east and southeast Europe. This encouraging data on gender parity in many eastern and southeastern European countries is a legacy of the Soviet ideology, which sought the emancipation of women. With the Soviet decrees of 1917, women no longer needed their husband's permission to work or study and over time, equal pay, maternity leave, and subsidised childcare arrangements were established. Although actual working conditions for women were nowhere near ideal, the Soviet Union achieved a high level of female representation in the workforce, including in STEM fields. Surprisingly, despite the introduction of these pioneering ideas, today women account for barely more than a third of researchers in Russia, by contrast to the countries that were part of the former Yugoslavia, where gender parity has been achieved. Women represent more than 50% of researchers in North Macedonia, Montenegro, Serbia, and Latvia and more than 45% in Lithuania, Croatia, Romania, and Bulgaria. Unfortunately, this is not the case in some high-income countries, such as France, Czech Republic, Germany, Luxembourg, and the Netherlands, where almost three-quarters of researchers are men.

Furthermore, the overall gender gap in Europe widens further as women progress up the scientific career ladder, representing a minority in top academic and decision making positions. This underrepresentation of women (aged ≥55 years) is a consequence of gender stereotyping in relation to care responsibilities and gender discrimination. For example, in only nine European countries (Belgium, Bulgaria, Denmark, Latvia, Luxembourg, Malta, Romania, Slovenia, and Iceland), women are more successful than men in obtaining research funding, which has a direct impact on their career development and promotion. In terms of exposure and recognition in the scientific community, women are underrepresented in scientific panels and conferences, they lag in the number of publications as they gain seniority, and are corresponding authors half as often as men. This gender gap is compounded by the fact that women are 21% less likely to be invited to write opinion pieces in their field of expertise. Additionally, women often experience maternal wall bias—ie, when colleagues view mothers or pregnant women as less competent and less committed to their jobs. Such bias is a major problem that hinders career advancement among women.

The cycle of poor recognition and unequal access to career opportunities that perpetuates the gender inequity gap has always existed and is now being accentuated by the COVID-19 pandemic. The Rapid Gender Assessment by UN Women, highlighted that “the pandemic is leading to backsliding in gender equality…and intensifying women's unpaid household and care work”. The time women have available for their research has decreased by 5% more than that for men, and for women with at least one child, this percentage increased to 17%. According to a survey by the Organization of Women in Science for the Developing World, almost 80% of female scientists reported spending more time than usual on household responsibilities during the COVID-19 pandemic, impacting the number of publications at all career levels. Between March 2019 and April 2020, a substantial decrease in the proportion of submissions by female senior researchers was observed and this decline is even more pronounced when focusing on first authors, who tend to be early career researchers.

Two years ago, the EU Commission launched the 2020-2025 Gender Equality Strategy, which envisions “a Europe where women and men, girls and boys, in all their diversity, are free from violence and stereotypes and have the opportunity to thrive and lead”. The strategy encourages women's participation in the labour market and a work-life balance, and promotes access to high quality and affordable childcare. A year later, in 2021, the EU Commission published a proposal for a law obliging companies to report gender pay gaps or face fines, in addition to rules to end gender discrimination. Although these important and much needed initiatives represent a shift in the right direction, revolutionary changes are not achieved through the implementation of public strategies alone, and require changes in beliefs and biases at the individual level. Implicit or unconscious gender bias, mainly secondary to the so-called science is male stereotype is known to play an important role in the failure to achieve gender parity, as noted in a Nature Human Behaviour publication. In this study, authors examined how a committee of French scientific evaluators decided which researchers should be promoted to elite positions. If the evaluators did not consciously believe in gender bias, they promoted fewer women than men, but if they recognised it, this tendency was reversed.

The first step towards gender equity is to acknowledge that gender bias exists and then to collectively work to eliminate the bias in our communities, workplaces, schools, colleges, and universities. This is not a commitment that we should honour only on International Women's Day, it should represent a life-long endeavour.

